# Treatment of Pyorrhœa Alveolaris

**Published:** 1899-07

**Authors:** John E. Heyke

**Affiliations:** University of Pennsylvania


### TREATMENT OF PYORRHŒA ALVEOLARIS.

BY JOHN E. HEYKE, UNIVERSITY OF PENNSYLVANIA.

The patient, a lady of fifty-nine years of age, had experienced no constitutional disorders for years, except a persistent neuralgia of the face. The gums looked very angry, showing deep recessions around the necks of the teeth, the pockets on the central incisors extending nearly to apex of root. No discharge of pus when pressing the gums. First treatment was given March 28, 1899. All deposits were removed and the parts cleansed with hydrogen dioxide, after which the affected tissues were thoroughly cauterized with a solution of sulphuric acid (twenty per cent.), afterwards neutralizing with sodium bicarbonate and packing the pockets with quinine, to prevent the cicatricial tissue forming from becoming invaded by bacteria. Central incisors were also widely separated.

Patient reported again after two weeks (April 11), when all the teeth affected exhibited considerable improvement, the central incisors not as much as the rest, which can probably be attributed to the lack of lateral support during mastication.

The hydrogen dioxide and quinine treatment were repeated, and, in addition, the central incisors were given a second application of sulphuric acid. The neuralgic pains had by this time disappeared altogether.

At the third visit, one week later, the cuspids and laterals were perfectly tight, and nothing was done to them; central incisors improving, and were treated with hydrogen dioxide and quinine. The fourth treatment was given the following week (April 25), when the central incisors were almost as firm as the rest.

Patient called again on May 2, when she was discharged, after having been advised to keep her teeth perfectly clean and to use a hydronaphthol mouth-wash, morning and night.

